# Combining Inertial Sensors and Machine Learning to Predict vGRF and Knee Biomechanics during a Double Limb Jump Landing Task

**DOI:** 10.3390/s21134383

**Published:** 2021-06-26

**Authors:** Courtney R. Chaaban, Nathaniel T. Berry, Cortney Armitano-Lago, Adam W. Kiefer, Michael J. Mazzoleni, Darin A. Padua

**Affiliations:** 1 Department of Exercise and Sport Science, University of North Carolina at Chapel Hill, Chapel Hill, NC 27599, USA; carmitan@email.unc.edu (C.A.-L.); awkiefer@email.unc.edu (A.W.K.); mmazzoleni@underarmour.com (M.J.M.); dpadua@email.unc.edu (D.A.P.); 2 Department of Kinesiology, University of North Carolina at Greensboro, Greensboro, NC 27402, USA; nathaniel.berry@underarmour.com; 3 Under Armour, Inc., Baltimore, MD 21230, USA

**Keywords:** biomechanics, jump landing, inertial sensors, machine learning, return to sport testing

## Abstract

(1) Background: Biomechanics during landing tasks, such as the kinematics and kinetics of the knee, are altered following anterior cruciate ligament (ACL) injury and reconstruction. These variables are recommended to assess prior to clearance for return to sport, but clinicians lack access to the current gold-standard laboratory-based assessment. Inertial sensors serve as a potential solution to provide a clinically feasible means to assess biomechanics and augment the return to sport testing. The purposes of this study were to (a) develop multi-sensor machine learning algorithms for predicting biomechanics and (b) quantify the accuracy of each algorithm. (2) Methods: 26 healthy young adults completed 8 trials of a double limb jump landing task. Peak vertical ground reaction force, peak knee flexion angle, peak knee extension moment, and peak sagittal knee power absorption were assessed using 3D motion capture and force plates. Shank- and thigh- mounted inertial sensors were used to collect data concurrently. Inertial data were submitted as inputs to single- and multiple- feature linear regressions to predict biomechanical variables in each limb. (3) Results: Multiple-feature models, particularly when an accelerometer and gyroscope were used together, were valid predictors of biomechanics (R^2^ = 0.68–0.94, normalized root mean square error = 4.6–10.2%). Single-feature models had decreased performance (R^2^ = 0.16–0.60, normalized root mean square error = 10.0–16.2%). (4) Conclusions: The combination of inertial sensors and machine learning provides a valid prediction of biomechanics during a double limb landing task. This is a feasible solution to assess biomechanics for both clinical and real-world settings outside the traditional biomechanics laboratory.

## 1. Introduction

Return to sport is of primary importance for a successful outcome following anterior cruciate ligament reconstruction (ACLR) [1]. However, athletes who do return to sport after ACLR are significantly more likely to sustain a second ACL injury compared to their previously uninjured counterparts [2,3,4], leading to inferior functional outcomes [5]. Hence, it is important to mitigate secondary injury risk through the identification and treatment of associated risk factors.

One proposed category of such risk factors is biomechanics and, specifically, biomechanical asymmetry. Asymmetrical biomechanics are often present following ACLR, with the involved limb exhibiting decreased peak vertical ground reaction force (vGRF), knee flexion angle (KFA), knee extension moment (KEM), and sagittal plane knee power absorption (KPA) during landing tasks compared to the contralateral limb [6,7,8]. Accordingly, the resolution of asymmetry is a common criterion for clearance to return to sport [9]. However, there are also differences in the contralateral limb relative to healthy controls [10,11]. This highlights the limitations associated with limb symmetry and provides opportunities to establish normative comparisons relative to previously uninjured athletes and to those who do not sustain secondary injuries.

Importantly, biomechanics during double limb landing tasks are also predictive of secondary ACL injury [12], including both graft tears [13] and contralateral injuries [14]. These changes include increased peak KFA as well as KEM and vGRF alterations throughout the landing phase relative to those who do not sustain a second ACL injury. These findings demonstrate that absolute measures of biomechanical variables (instead of limb symmetry) are particularly important for secondary ACL injury risk. Additionally, targeted neuromuscular training is effective in improving biomechanical deficits at the knee in individuals post-ACLR [15], and such interventions may decrease secondary injury risk [16]. Given the prevalence, predictive capability, and responsiveness to intervention, the resolution of biomechanical impairments should be a key target during rehabilitation [17].

Despite the importance of biomechanics within this paradigm, most clinicians lack access to clinical solutions to objectively assess biomechanics. The gold-standard three-dimensional motion capture and force plates used to index the previously mentioned deficits are only available in select laboratory settings. This is because they are expensive, require trained experts to administer, and require considerable time for set-up and processing, all of which preclude wide-spread adoption. In contrast, inertial sensors are inexpensive and portable, both of which address barriers associated with biomechanical evaluation and clinical implementation. As commercially-available inertial sensor systems with the capability to quantify external loading during sports-related activities become more widely available [18], inertial sensors are being used to quantify biomechanics with increasing frequency [19]. Most of these systems provide measures of global lower extremity loading, such as peak tibial acceleration, which correlates with vGRF metrics [20,21]. While often global (e.g., peak vGRF) and knee-specific (e.g., KEM) measures of loading demonstrate impaired biomechanics during landing tasks post-ACLR [7,22], indexing only global measures of loading can cause misrepresentations of tissue-specific loading [23]. Therefore, in order for inertial sensor solutions to provide optimal information to augment clinical decision-making in rehabilitation post-ACLR, they should provide surrogates for both global and knee-specific loading.

Single variable inertial sensor solutions (e.g., peak angular velocity from one axis of a gyroscope or peak acceleration from one axis of an accelerometer) provide surrogates of knee-specific biomechanics during landing tasks both in healthy [24] and ACL-injured participants [25,26]. Additionally, the use of inertial sensors combined with machine learning to predict biomechanics in musculoskeletal-injured populations has grown exponentially in recent years [19], serving as a promising interdisciplinary solution. This combined approach has proven successful for predicting knee-specific biomechanics for single-limb tasks including running, jump landing, and cutting [27,28,29]. However, these solutions have not specifically targeted double limb landing tasks, which have clinical relevance post-ACLR.

In order to address the clinical need for a simple, objective solution to quantify biomechanics, this project seeks to combine inertial sensors and machine learning to predict both global (peak vGRF) and knee-specific (peak KFA, KEM, and KPA) biomechanical variables during a double limb landing task. We selected this task due to its recommended use in return to sport testing [30] and its utility for the accurate prediction of secondary injury risk [12,13,14]. The purposes of this study are, thus, to: (1) develop multi-sensor machine learning algorithms for predicting biomechanics and (2) quantify the accuracy of each algorithm. In order to provide context for our findings, we will concurrently present single feature algorithms, which enable us to make recommendations for optimal solutions in a clinical rehabilitation setting.

## 2. Materials and Methods

### 2.1. Overview

**Figure 1:**
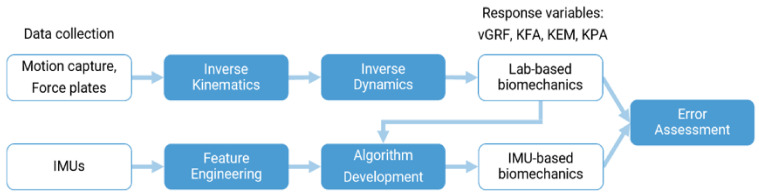
Overview of project. For the calculation of laboratory-based biomechanics, we collected motion-capture and force plates data. We used inverse kinematics and inverse dynamics to calculate vGRF (peak vertical ground reaction force), KFA (peak knee flexion angle), KEM (peak knee extension moment), and KPA (peak sagittal plane knee power absorption). For the modeling of inertial measurement unit (IMU)-based biomechanics, we collected IMU data concurrently. We then selected the region of interest of these time series and extracted features (feature engineering). Next, we developed algorithms to predict the lab-based biomechanics. We evaluated the error of the IMU-based biomechanics against the lab-based biomechanics.

To conduct this project, we simultaneously collected motion capture, force plate, and inertial measurement unit (IMU) data while participants completed a jump-landing task. We used inverse kinematics and inverse dynamics to calculate our laboratory-based biomechanical variables of interest based on the gold-standard of motion capture and force plate signals. Next, we used the IMU signals to predict the laboratory-based biomechanical variables. To do so, we extracted IMU features (e.g., maximum and minimum acceleration) then fit both simple and multiple regression models. We evaluated the performance of these models against the laboratory-based values. A diagram outlining this process is depicted in Figure 1.

### 2.2. Participants

Twenty six healthy college students (25 female) participated in the study. Participants averaged 20.0 ± 1.3 years of age, 171 ± 8 cm tall, and 68.8 ± 10.3 kg of mass. Inclusion criteria included self-reported participation in moderate physical activity at least three times per week for 30 min or more, per the guidelines of the American College of Sports Medicine. Exclusion criteria included any lower extremity injury within the last six months. This study was approved by our university’s institutional review board, and all participants provided written informed consent for participation.

All participants wore black spandex and standard lab shoes (Under Armour HOVR Sonic). We taped retroreflective markers over bony landmarks and segments of each participant. We used 7 clusters of 3-4 markers each, which we placed over the sacrum, bilateral lateral thighs, lateral shanks, and dorsal feet. We used 9 additional tracking markers over the following landmarks: C7, sternum, L4/5, bilateral acromion, anterior superior iliac spine (ASIS), and posterior calcaneus. We used an additional 10 markers for the static calibration trial over the following landmarks: bilateral greater trochanters, medial and lateral femoral epicondyles, 1st and 5th metatarsal heads.

**Figure 2:**
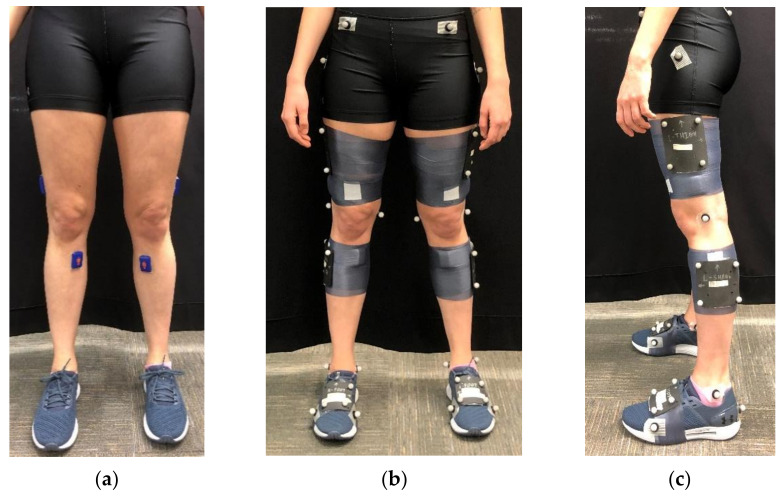
Pictures of marker and IMU configurations. (**a**) IMU placement; (**b**) Frontal view of full marker configuration; (**c**) Sagittal view of full marker configuration.

We adhered two IMUs (Blue Trident, Vicon, Nexus, Oxford, UK) on each limb with double-sided tape and secured the IMUs with pre-wrap and cloth tape. We placed the thigh IMU on the distal thigh, directly lateral over the iliotibial band. The bottom of the IMU was 8 cm proximal to the tibiofemoral joint line. We placed the shank IMU on the flat aspect of the proximal anteromedial tibia. The top of the IMU was 5 cm distal to the tibiofemoral joint line. Both IMUs were oriented so that the positive *x*-axis was pointing superiorly. We selected these IMU locations to minimize soft tissue artefact and for their proximity to the knee for potential incorporation into knee braces, similar although not identical to Stetter et al. [28]. We ensured that the thigh and shank marker clusters were not in contact with the IMUs. Pictures of marker and IMU configurations can be seen in Figure 2.

### 2.3. Data Collection

All participants performed eight trials of a double-limb jump landing task as previously described [31,32]. We instructed participants to jump forward from a 30 cm tall box to side-by-side embedded force plates then complete a maximal vertical jump immediately upon landing. The distance from the box to the force plates was half of the participant’s height. We deemed a trial successful if the participant (1) jumped forward with both feet to reach the force plates, (2) jumped vertically during the maximal jump, and (3) completed the task in a fluid motion. We gave participants a minimum of two practice trials prior to data collection.

Three-dimensional coordinates of retroreflective markers were collected at a sampling frequency of 250 Hz using a 10-camera motion capture system (Vicon, Nexus, Oxford, UK). The *x*-axis was pointing forward, *y*-axis was pointing toward the left, and *z*-axis was pointing upward. Ground reaction forces were collected at a sampling frequency of 1000 Hz from two embedded force plates (FP406020, Bertec Corp, Columbus, OH, USA). IMU data were collected at a sampling frequency of 1125 Hz including dual-g accelerometers (high: ±200 g, low: ±16 g), gyroscope (±2000°/s), and magnetometer (±4900 µT). All data were time-synchronized and collected in Nexus software (v2.10, Vicon, Oxford, UK).

### 2.4. Laboratory-Based Biomechanical Analysis (Motion Capture and Force Plates)

We fit all time-series data with linear interpolation and resampled at 1250 Hz. We used a fourth order, 15 Hz low-pass Butterworth filter for marker trajectories and force plate data that were used in joint moment calculations [33,34]. Additionally, we used a fourth order, 100 Hz low-pass Butterworth filter for force plate data used to calculate the peak vGRF.

The hip joint center was defined using the Bell method [35]. The knee joint center was defined as the midpoint between the lateral and medial femoral epicondyles. The ankle joint center was defined as the midpoint between the lateral and medial malleoli. An inverse kinematics approach was used to calculate Cardan angles between thigh and shank segments in an order of flexion-extension, abduction-adduction, and internal-external rotation. A standard inverse dynamics approach was used to calculate joint moments and power of the distal segments relative to proximal segments. Moments were expressed as internal moments. These tasks were completed in Visual 3D software (C-Motion Inc., Rockville, MD, USA).

**Figure 3:**
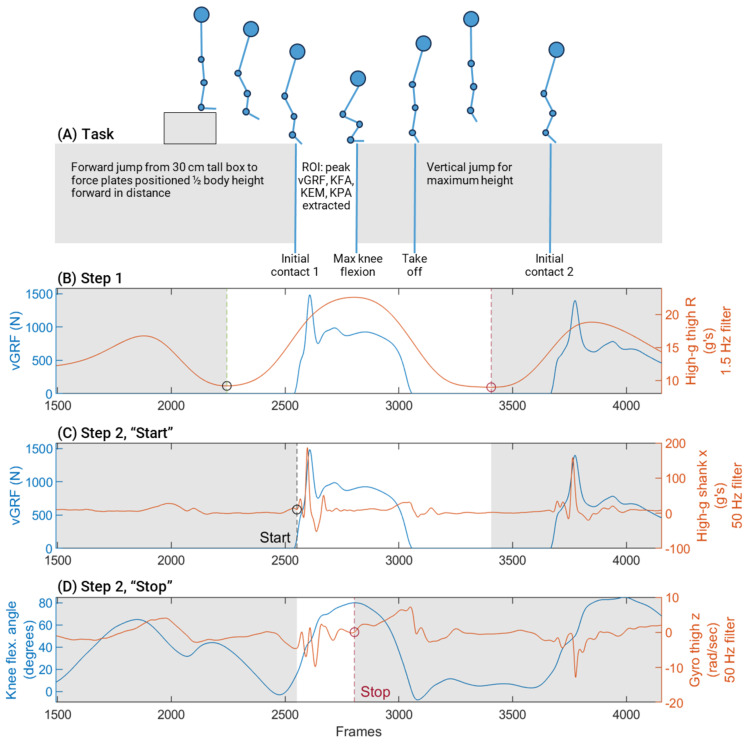
Description of task and steps to select region of interest (ROI)-based on IMUs. (**A**) Task. Participants jumped forward from a 30 cm tall box to side-by-side force plates positioned ½ body height forward in distance. Immediately upon landing, they completed a maximum vertical jump and landed back on the force plates. The region of interest to extract biomechanical variables was from initial contact of the first landing until maximum knee flexion during that landing. (**B**) Step 1. Identification of the initial ROI based on the 2 most prominent local minima of the resultant thigh acceleration after applying a 1.5 Hz low-pass filter. Circles indicate these two points. (**C**). Step 2, “Start.” Identification of the “start” within the ROI from step 1, based on the local minimum immediately preceding when the high-g shank x signal crossed 20 g’s. A black circle indicates this point. (**D**). Step 3, “Stop.” Identification of the end of the ROI when the thigh gyroscope data was greater than 0 for at least 50 frames. A red circle indicates this point. vGRF and knee flexion angles are overlaid to show that the ROI targeted the first half of the landing, from approximate initial contact to maximum knee flexion angle. IMUs, inertial measurement units. ROI, region of interest. vGRF, peak vertical ground reaction force. KFA, peak knee flexion angle. KEM, peak internal knee extension moment. KPA, peak sagittal plane knee power absorption. BW, body weight. HT, height.

We selected one response variable to represent overall limb loading during landing: peak vGRF (normalized by body weight in N). We selected three response variables to represent the sagittal plane kinematics and kinetics of the knee during landing: peak KFA in degrees, peak internal KEM in Nm and normalized by the product of body weight in N and height in m, and peak KPA in watts, normalized by the product of body weight in N and height in m. All variables were extracted from time-series data as the maximum (or minimum) values during the landing phase of the first jump from the frame of initial contact (when the vGRF first exceeded 10 N) until maximum knee flexion. A visualization of this region can be seen in Figure 3A. KPA values, which were negative by definition, were multiplied by −1 for ease of interpretation.

### 2.5. Inertial Measurement Unit (IMU)-Based Biomechanical Analysis

We processed all IMU data and completed model training with custom MATLAB scripts (v2019a, The Mathworks Inc., Natick, MA, USA).

#### 2.5.1. Region of Interest

We examined time-series GRF and IMU signals for each limb-trial with a goal of defining a region of interest (ROI) from the IMUs alone. Our goal was for this region to coincide with the landing phase (from initial contact to maximum knee flexion) after the first jump. While the IMU ROI could be detected with assistance from the force plate data, we considered that the ability to determine the ROI from the IMU data alone was critical to make our algorithms clinically feasible. Ultimately, we accomplished this through two steps:

1. We corrected all right limb IMUs to mirror the axes of the left limb IMUs. We applied a second-order, 1.5 Hz low-pass Butterworth filter [36] on the thigh high-g accelerometer time-series for all three axes and then calculated the resultant acceleration. We found the two most prominent [37] local minima of the resultant acceleration and defined the initial ROI as the region between these two points. An example trial can be seen in Figure 3B.
2. To further refine the ROI, we used the selected region from step one, then determined “start” and “end” points within this region. Since optimal filtering parameters of inertial sensors during landing tasks have not been established, we explored a range of low pass filtering parameters from 15 Hz [28] up to unfiltered. Ultimately, we elected to apply a second-order, 50 Hz low-pass Butterworth filter on all IMU time-series data. This filter allowed for reliable feature extraction while visually appearing to reduce high frequency noise. The start point occurred at the local minimum directly preceding when the shank x (aligned axially on the shank) high-g accelerometer first exceeded 20 g’s for five consecutive frames (4 ms). The end point occurred after the start point, when the thigh z (aligned with the medial-lateral axis of the thigh) gyroscope exceeded 0 rad/sec for at least 50 frames (40 ms) forward, indicating angular velocity of the thigh towards relative extension. All trials were visually inspected with overlaid vGRF and KFA to ensure these steps yielded an appropriate region. A visualization of these steps for a representative limb-trial is shown in Figure 3C,D.

#### 2.5.2. Feature Engineering

Consistent with step two in selecting the ROI, we used a second order, 50 Hz low-pass Butterworth filter on IMU time-series data and then calculated resultants for each signal. Next, we extracted features from each high-g accelerometer and gyroscope time-series (*x* axis, *y* axis, *z* axis, and the resultant) within the ROI. A full list of the 14 features extracted from each time-series along with definitions can be found in Table 1. We used the length of time (range) of the ROI as an additional feature for each IMU. This yielded 225 features (14 features × 8 time-series × 2 IMUs + 1 ROI range = 225) for each limb per trial. After feature extraction, we normalized all features to center at 0 with a standard deviation of 1 prior to model training.

#### 2.5.3. Algorithm Development

For a summary of model characteristics, including input parameters, model training, model selection, and performance evaluation, see Table 2. We collapsed trials across limbs for analysis. Each participant had eight right limb-trials and eight left-limb trials, yielding 416 total limb-trials across all participants. To reference single-sensor, single-feature IMU algorithms that are either available commercially or presented in research [24,26], we performed simple linear regression between each predictor variable and response variable for each task, then we selected the predictor variable from each IMU with the highest R^2^. These models are referred to as “single feature shank” and “single feature thigh.” We then used machine learning to fit multiple-sensor, multiple-feature models using stepwise linear regressions for all accelerometer features (“multiple feature accel”) and all accelerometer and gyroscope features (“multiple feature accel + gyro”). These combinations were selected due to some clinically available sensors containing only accelerometers, while others contain both accelerometers and gyroscopes. We allowed constant and linear terms in these models. We utilized hyperparameter optimization by performing a grid search on the criteria for terms to be added or removed from the models. Specifically, we set a range of thresholds for the *p*-value of an *F*-test of the change in the sum of squared error (SSE) resulting from adding or removing a term from the model. We selected the final models based on maximizing R^2^ while minimizing the number of features required, with a target of no more than 41 features (1 feature per 10 limb-trials). This technique was used to avoid overfitting our models [38]. For each selected multi-feature model, we also performed *k*-fold cross-validation (n = 10) by randomly assigning each limb-trial to 1 of 10 folds. Based on this random fold-assignment, individual participant limb-trials were dispersed throughout multiple folds. We trained models on 9 folds then tested on the remaining fold and repeated this process across each fold. We then calculated the mean and standard deviations of R^2^, root mean square error (RMSE), and normalized root mean square error (nRMSE) across all folds.

#### 2.5.4. Algorithm Evaluation

For each selected model, we calculated the coefficient of determination (R^2^) across all trials. Since there is no widely accepted threshold of R^2^ in exploratory research such as this, we referenced similar algorithm development [39], which used R^2^ > 0.80 as high algorithm accuracy. We calculated the RMSE to present the error of the model in absolute terms in the units of measurement. We then calculated the nRMSE by dividing the RMSE over the range (maximum–minimum values across all participants) of the data. The nRMSE allows relative comparisons of percent model error between models with different units, thus allowing us to compare the relative error between response variables.

## 3. Results

A summary of descriptive data on the four response variables, including mean values by trial can be found in Table 3. The distribution of KPA was significantly positively-skewed, with three limb-trials greater than the mean + 3.5 standard deviations in magnitude (average *z*-score of 4.5). We considered these to be outliers and removed these limb-trials from the KPA models. We calculated within-participant variation by calculating each participant’s standard deviation across all trials from both limbs then averaging across all participants. We calculated between-participant variation by calculating each participant’s mean across all trials then calculating the standard deviation between participants.

**Figure 4:**
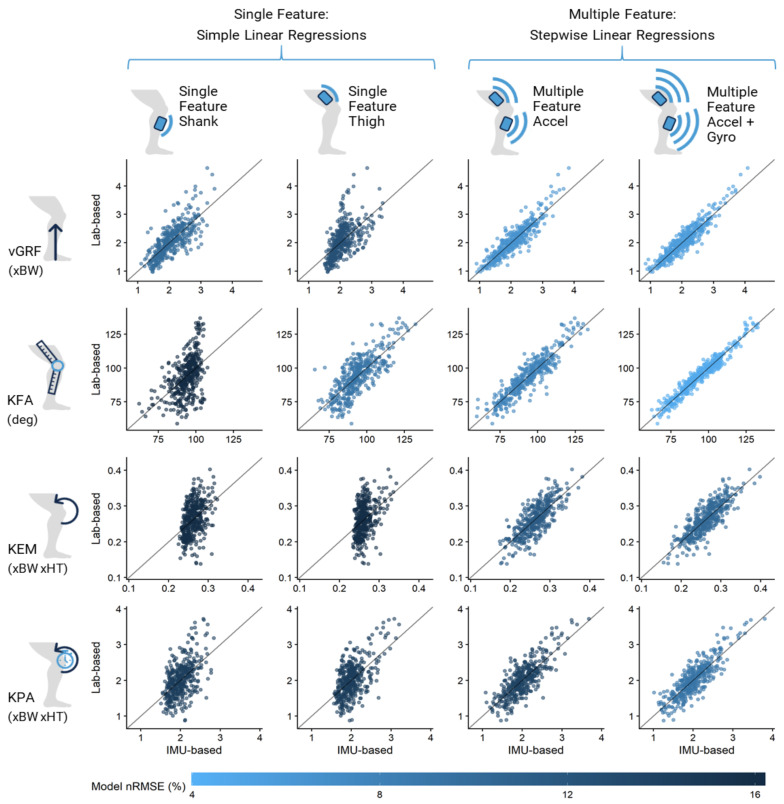
Scatter plots of expected (based on motion capture and force plates) vs. predicted (based on IMUs) values for each model by response variable. Each dot represents a limb-trial. Dots are colored according to the average nRMSE (normalized root mean square error) of the model, meaning darker colored models had a higher percent of normalized error, while lighter colored models had a lower percent of normalized error. vGRF, peak vertical ground reaction force. KFA, peak knee flexion angle. KEM, peak internal knee extension moment. KPA, peak sagittal plane knee power absorption. BW, body weight. HT, height.

Scatterplots depicting the fit and nRMSE of each selected model can be seen in Figure 4, and additional details regarding the accuracy, error, and cross-validation of the models can be found in Table 4. Cross-validation resulted in minimal increases in model prediction error, suggesting that our models were not overfit. Tables of all features used in each selected model can be found in Appendix A.

## 4. Discussion

Our findings indicate that leveraging data science approaches in combination with inertial sensors yields valid predictions of global and knee-specific loading during a double-limb landing task. Additionally, the methods employed in this project lend themselves to clinical implementation by reducing barriers of cost, set-up time and space, as well as processing time. We will contextualize our findings and discuss opportunities for advancement and implementation.

### 4.1. Overview of Findings

We first sought to understand if the degree of error in our algorithms was acceptable to recommend their use. To provide clinical context of the error in our models, we compared the RMSE of each model to prior research that reported the difference in means between the involved limb post-ACLR and healthy control limbs during the same double limb landing task. In the case of multiple studies reporting these variables, we used the one with the largest sample size. For KPA only, we were unable to identify prior work based on our defined criteria. Instead, we referenced White et al. [40], who reported a single limb landing task. We scaled the reported joint powers due to differing normalization so that the reported mean in the healthy controls (17.21) was equivalent to our mean of 2.01.

To interpret these findings, we used the following guidelines: If the clinical difference was larger than our error, we could be more confident in recommending use of the algorithm. In contrast, if the clinical difference was smaller than our error, the algorithm may not function at a sufficient level of accuracy. The results of this comparison can be seen in Table 5. Across vGRF, KFA, KEM, and KPA, both multiple feature models had smaller RMSE compared to the clinical difference, supporting their use. In contrast, both single feature models had larger RMSE compared to the clinical difference, limiting their clinical utility.

When considering the combination of model goodness of fit (R^2^) and clinical context of error, we suggest that all multiple feature models presented are valid predictors of biomechanics, with the addition of the gyroscope improving models across all variables. While the R^2^ for the KEM and KPA models did not exceed the benchmark of 0.8 for high accuracy, the error did not exceed the expected differences between ACLR and healthy control limbs, hence there is still clinical value in these models.

Models may not extrapolate well to data outside the range of the data on which they were trained. Across all response variables, the range of our data included the mean values observed in ACLR limbs. However, our range likely did not include all possible values observed in ACLR limbs. Our models would be strengthened by the addition of a wider range of values, including those from ACLR limbs. Prior to advocating for clinical use of our models, we suggest additional model training that includes the population of interest. We anticipate that these models would have similar error, but they would also have improved accuracy on data outside the range of the current dataset.

### 4.2. Absolute vs. Relative Measures of Biomechanical Variables

We advocate for absolute measures of biomechanical variables as opposed to the relative symmetry of these variables between limbs. Absolute measures of biomechanical variables are predictive of secondary ACL injury [13,14], while the utility of limb symmetry indices has been called into question [10]. Our algorithms were developed with the goal of predicting absolute measures of biomechanical variables. Given that limb symmetry is currently utilized clinically, but not a focus of our project, we have included a symmetry analysis in Appendix B.

### 4.3. Comparing and Contrasting Double and Single Limb Landings

Most of the projects we identified that have used inertial sensors to predict knee-specific biomechanics have analyzed single limb landing tasks, in contrast to the double limb landing task we assessed. Double limb landing tasks have more capacity for asymmetry in GRF due to their bipedal nature, which in turn influences joint-specific kinetic variables, including those at the knee. Thus, the accuracy of predicting biomechanics during this task appears to be more challenging. Stetter et al. [28] used inertial sensors in combination with an artificial neural network (ANN) to predict knee joint forces and reported decreased model accuracy during double limb landings in comparison to single leg landings. They suggested using an activity-recognition approach to improve model accuracy, which is feasible when performing tasks as a part of a return to sport battery.

The variance in loading during double limb tasks is corroborated in athletes after ACLR, in whom differences are observed in peak vGRF and peak KEM for double but not single limb landings [6]. This may help explain why double limb landing mechanics are predictive of secondary ACL injury risk [13,14], despite the primary mechanism of ACL injury being largely during single limb loading [44]. Combined, these findings support that while there are challenges of accurately modeling double limb landings due to force distribution variability between limbs, it is important to identify solutions that are able to do so.

### 4.4. Benefits and Drawbacks of Single-Feature vs. Multiple-Feature Solutions

We presented single-feature models alongside the multiple-feature models in order to reference currently available algorithms. There is considerable appeal to the simplicity of using one feature (e.g., a maximum value) to provide surrogate information regarding biomechanical parameters of interest. Accordingly, there has been success in using this approach, particularly with single limb tasks. At the knee, specifically, Morgan et al. [24] used a shank accelerometer for single leg landings during “preferred”, “soft”, and “stiff” conditions and found a strong correlation between peak posterior acceleration and peak KEM in healthy participants (R^2^ = 0.76). Pratt et al. [26] used a thigh gyroscope for single leg landings in participants post-ACLR and found correlations between peak thigh angular velocity and peak KPA (r = 0.81) and peak KEM (r = 0.59).

Real time biofeedback interventions using single features have successfully altered both inertial sensor measures (angular velocity and accelerations) as well as biomechanics assessed through traditional laboratory measures. Specific to jump landing tasks, Dowling et al. [45] altered both thigh angular velocity and knee abduction moment through inertial sensor-driven feedback. There is also a body of literature demonstrating the effectiveness of modifying GRF loading rates during running using single features from shank accelerometers [46,47,48].

Despite these successful feedback interventions, we emphasize that our multiple feature models, for knee kinetics in particular, had large improvements in accuracy and decreases in error compared to our single feature models: KEM R^2^ improved from 0.17 at best to 0.68, with a decrease in nRMSE from 15.9% to 10.2%, and KPA R^2^ improved from 0.34 at best to 0.72, with a decrease in nRMSE from 13.7% to 9.12%. While there is still error in these models, they provide superior predictive capabilities.

Based on our findings and prior research, we draw two conclusions regarding single vs. multiple feature models:

1. We recommend that the use of single features is ideally suited for feedback interventions, and we advocate for future interventional research to demonstrate the effectiveness of manipulating knee-specific biomechanics post-ACLR.
2. We recommend the use of multiple feature models for improved fidelity in objectively assessing biomechanics during landing tasks outside a laboratory setting.

### 4.5. Machine Learning Approaches

In order, the most commonly reported machine learning algorithms utilized in human movement biomechanics are support vector machines (SVMs), ANNs, and generalized linear models, such as our project [19]. Specific to knee biomechanics, Stetter et al. used IMUs on the thigh and shank combined with ANNs to predicted knee joint forces [28] and moments [27] across a variety of athletic maneuvers. The only double limb task used was a two-leg jump, and reported percent difference between the expected and predicted peak vertical knee force was 22.9%, highlighting the challenges of estimating double limb tasks. Gholami et al. [29] used a single accelerometer on the foot combined with a convolutional neural network (CNN) to predict lower extremity kinematics during running gait, but did not predict kinetics. In relation to the work cited above, our project strategy specifically targeted the task and biomechanical variables of interest in post-ACLR. Additionally, the stepwise linear regressions that we used are straightforward to implement and less susceptible to overfitting than other methods, which we view as a strength for clinical translation. We recommend that future work explore additional machine learning algorithms, such as support vector machines or neural networks.

### 4.6. Variability of Landing Strategy

As this was a proof-of-concept approach to predict absolute measures of loading, we did not manipulate participants’ preferred landing strategies. For future work, we recommend systematic manipulation of landing (e.g., through instructions to “land softer” or “land stiffer”) in addition to the inclusion of participants post-ACLR. This will result in greater within-subject variability and will also increase the range of data on which the algorithm is trained. It will also provide understanding as to the degree to which the algorithm can assess change within individuals, which will be important when implementing interventions aimed to alter biomechanics. This, in addition to establishing the between-day reliability of these algorithms, will be important foundational knowledge necessary to support the utilization of IMUs for interventions and serial assessments.

### 4.7. Additional Considerations

There are several important considerations when selecting inertial sensors to be used in a similar manner. The inertial sensors we utilized in this project are marketed for both research and clinical purposes, thus we believe sensors such as these to be a clinically feasible solution. Based on the parameters identified in this project, we suggest that sampling frequency and accelerometer range are important to consider during jump landing tasks given both the speed of the task and acceleration generated from ground impact. We did not use the low-g accelerometer in our analyses because the range of this accelerometer was often exceeded upon initial contact. Furthermore, our sensors were aligned and oriented based on anatomical landmarks. Since features from most axes were included in our selected multiple feature models (see Appendix A for details), the models will be sensitive to the sensor orientation. Care should be taken to mount and fix the sensors as described. Gyroscope drift is another consideration, as it can create significant error during longer duration tasks. However, we do not expect drift to have a strong influence on data fidelity for a short duration jump landing task.

## 5. Conclusions

In summary, we combined data science techniques with inertial sensors to predict global and knee-specific biomechanics during a double limb landing task. This work provides a simple clinical solution to objectively quantify biomechanics. Our multiple feature algorithms, particularly with the addition of the gyroscope, were valid predictors of biomechanics (normalized error ranging between 4.6–10.2%). Additionally, all multiple feature model errors were lower than the clinical differences between ACLR involved limbs and healthy control limbs, further supporting the utility in this patient population. Future research should focus on increasing variability in landing strategy, testing on more varied populations (including patients), and establishing between-day reliability.

## Figures and Tables

**Table 1:** Features extracted from each time-series ROI.

Category	Variable Name	Calculation
Max	Max	Maximum value
	Time to max	Frame number at maximum value
	Max prominence ^1^	Height of maximum relative to surrounding time-series
	Width of max	Width (number of frames) at half-prominence ^1^
Min	Min	Minimum value
	Time to min	Frame number at minimum value
	Min prominence ^1^	Height of minimum relative to surrounding time-series
	Width of min	Width (number of frames) at half-prominence ^1^
Max-min	Max-min difference	Maximum value–minimum value
	Max-min time difference	Time to max–time to min
Other	Start value	Value at start of ROI
	Stop value	Value at end of ROI
	Standard deviation	Standard deviation of all elements
	Area under the curve	Approximate integral using trapezoidal numerical integration

^1^ For a detailed description of the prominence calculation, see [37]. ROI, region of interest.

**Table 2:** Algorithm development, selection, and performance evaluation.

		Model			
		Single Feature		Multiple Feature	
		Shank	Thigh	Accel	Accel + Gyro
Model input parameters	Sensor location(s)	Shank	Thigh	Shank and thigh	
	Signals	Accel, gyro		Accel	Accel, gyro
	Potential features	113	113	113	225
Model training and selection	Model used	Simple linear regression		Stepwise linear regression	
	Hyperparameter optimization	No		Yes	
	# of selected features	1		Up to 41	
	Model selection	Highest R^2^		High R^2^, low # of features	
	Cross-validation	No		Yes, *k*-fold, n = 10	
Performance evaluation	Goodness of fit	R^2^		R^2^	
	Error	RMSE, nRMSE		RMSE, nRMSE	

RMSE, root mean square error. nRMSE, normalized root mean square error.

**Table 3:** Summary data on response variables. N represents the number of limb-trials.

Variable	N	Mean ± SD	Range [Min, Max]	Mean within- Participant SD	Mean between-Participant SD
vGRF (xBW)	416	2.07 ± 0.57	[0.96, 4.63]	0.32	0.48
KFA (deg)	416	93.9 ± 14.4	[58.9, 136.9]	5.0	13.8
KEM (xBW xHT)	416	0.262 ± 0.046	[0.138, 0.402]	0.033	0.031
KPA (xBW xHT)	413	2.01 ± 0.48	[0.87, 3.72]	0.33	0.38

vGRF, peak vertical ground reaction force. KFA, peak knee flexion angle. KEM, peak internal knee extension moment. KPA, peak sagittal plane knee power absorption. BW, body weight. HT, height.

**Table 4:** Model prediction, accuracy, and cross-validation by response variable.

		Model				Cross-Validation	
		Single Feature		Multiple Feature		Multiple Feature	
		Shank	Thigh	Accel	Accel + Gyro	Accel	Accel + Gyro
vGRF (xBW)	Features (#)	1	1	21	27		
	R^2^	0.58	0.36	0.82 *	0.87 *	0.78 ± 0.01	0.83 ± 0.01
	RMSE	0.37	0.46	0.24	0.21	0.25 ± 0.003	0.22 ± 0.002
	nRMSE (%)	10.0	12.5	6.5	5.7	6.8 + 0.08	6.0 ± 0.05
KFA (deg)	Features (#)	1	1	23	41		
	R^2^	0.24	0.60	0.83 *	0.94 *	0.80 ± 0.01	0.92 ± 0.003
	RMSE	12.6	9.1	6.1	3.6	6.2 ± 0.05	3.8 ± 0.04
	nRMSE (%)	16.2	11.7	7.8	4.6	7.9 ± 0.06	4.9 ± 0.05
KEM (xBW xHT)	Features (#)	1	1	24	31		
	R^2^	0.17	0.16	0.59	0.68	0.50 ± 0.01	0.60 ± 0.01
	RMSE	0.042	0.042	0.030	0.027	0.031 ± 0.0002	0.028 ± 0.0002
	nRMSE (%)	15.9	15.9	11.4	10.2	11.7 ± 0.07	10.6 ± 0.07
KPA (xBW xHT)	Features (#)	1	1	30	33		
	R^2^	0.27	0.34	0.63	0.72	0.53 ± 0.02	0.64 ± 0.01
	RMSE	0.41	0.39	0.30	0.26	0.32 ± 0.003	0.27 ± 0.003
	nRMSE (%)	14.3	13.7	10.5	9.1	11.2 ± 0.1	9.5 ± 0.1

* Indicates R^2^ values greater than or equal to 0.80, the benchmark for high accuracy. Cross-validation includes the mean ± standard deviation across all folds. vGRF, peak vertical ground reaction force. KFA, peak knee flexion angle. KEM, peak internal knee extension moment. KPA, peak sagittal plane knee power absorption. BW, body weight. HT, height. RMSE, root mean squared error. nRMSE, normalized root mean squared error.

**Table 5:** Model error compared to mean clinical difference in anterior cruciate ligament reconstruction (ACLR) subjects vs. healthy controls.

	Prior Research				Current Models (RMSE)			
					Single Feature		Multiple Feature	
Variable	Reference	ACLR Involved	Healthy Control	Diff.	Shank	Thigh	Accel	Accel + Gyro
vGRF	Paterno et al. [41]	1.77	2.01	0.24	0.37	0.46	**0.24**	**0.21**
KFA	Delahunt et al. [42]	62.0	69.5	7.5	12.6	9.1	**6.1**	**3.6**
KEM	Goerger et al. [43]	0.169	0.204	0.035	0.042	0.042	**0.030**	**0.027**
KPA	White et al. [40]	1.65	2.01	0.36	0.41	0.39	**0.30**	**0.26**

Bold indicates RMSE values that are ≤ mean clinical difference. vGRF, peak vertical ground reaction force. KFA, peak knee flexion angle. KEM, peak internal knee extension moment. KPA, peak sagittal plane knee power absorption. RMSE, root mean squared error.

**Figure A1:**
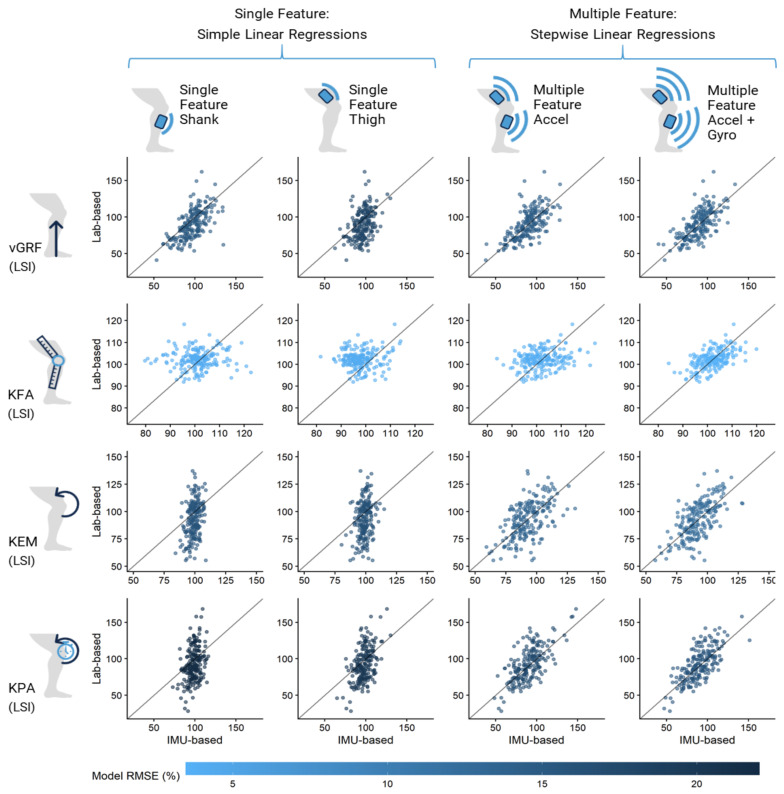
Scatter plots of expected (based on motion capture and force plates) vs. predicted (based on IMUs) limb symmetry index (LSI) values for each model by response variable. Each dot represents a limb-trial. Dots are colored according to the average RMSE (root mean square error) of the model, meaning darker colored models had higher error, while lighter colored models had a lower error. vGRF, peak vertical ground reaction force. KFA, peak knee flexion angle. KEM, peak internal knee extension moment. KPA, peak sagittal plane knee power absorption.

## Data Availability

Not Applicable.

## Appendix A

We have included a full list of features included in each model. The abbreviations for all feature names are included in Table A1. The features used in single feature models are listed in Table A2. The features used in multiple feature accel models are in Table A3. The features used in multiple feature accel and gyro models are in Table A4.

When examining single feature models, all models used features were from the accelerometer, with the exception of thigh KFA, which used a gyroscope feature. When further exploring the features used in multiple feature models, the top five features (as defined by the highest absolute values of coefficients) always included both thigh and shanks sensors, suggesting that the combination of both sensors may be important for high algorithm accuracy across variables.

**Table A1:** Abbreviations for feature names.

Feature Name	Abbreviation
Max	max
Time to max	ttmax
Max prominence	pmax
Width of max	wmax
Min	min
Time to min	ttmin
Min prominence ^1^	pmin
Width of min	wmin
Max-min difference	mmdiff
Max-min time difference	mmtdiff
Start value	start
Stop value	stop
Standard deviation	std
Area under the curve	auc

**Table A2:** Variables used in single feature models.

Response	Shank	Thigh
vGRF	Accel R: std	Accel R: std
KFA	Accel Z: std	Gyro Z: auc
KEM	Accel X: std	Accel Y: std
KPA	Accel X: std	Accel Y: std

For variable abbreviations, see Table A1. vGRF, peak vertical ground reaction force. KFA, peak knee flexion angle. KEM, peak internal knee extension moment. KPA, peak sagittal plane knee power absorption. RMSE, root mean squared error.

**Table A3:** Features used in multiple feature accel models.

Response	Shank Accel	Thigh Accel	Other
vGRF	X: start, **auc** Y: **max**, pmax Z: wmax, min R: stop, **std**, **auc**	X: **auc** Y: start, ttmax, pmin, auc Z: start, pmax, wmin, auc R: start, stop, mmdiff	
KFA	X: start, **std**, min, pmin, mmtdiff, **auc** Y: start Z: **std**, ttmin, mmdiff R: start, stop	X: wmin, auc Y: min, wmin, **auc** Z: start, pmin, mmdiff R: auc	**Range**
KEM	X: start, ttmax Z: start, w max R: std, **max**, ttmax, min	X: **std**, wmax, **mmdiff**, **auc** Y: start, wmax, auc Z: wmax, pmax, **mmdiff**, mmtdiff, auc R: start, ttmax, wmin, auc	
KPA	X: min, ttmin, pmin, mmtdiff Y: ttmax, wmin, auc Z: wmax, pmax, mmtdiff R: **std**, **max**, wmax	X: auc Y: start, **std**, **max**, **pmax**, pmin Z: start, std, ttmax, pmax, wmin, auc R: start, wmax, pmax, ttmin, pmin	

Bolded variables are the top 5 features from each selected model based on the highest absolute value of model coefficients. For variable abbreviations, see Table A1. vGRF, peak vertical ground reaction force. KFA, peak knee flexion angle. KEM, peak internal knee extension moment. KPA, peak sagittal plane knee power absorption. RMSE, root mean squared error.

**Table A4:** Features used in multiple feature accel + gyro models.

	Shank		Thigh		
Response	Accel	Gyro	Accel	Gyro	Other
vGRF	X: start, ttmin, auc Y: std, min R: start, **std**, ttmax	Y: start, std, pmax, ttmin Z: ttmin R: **start**, **max**, **pmax**	Y: pmin Z: wmin, pmin, auc R: stop	X: ttmax, wmax Y: mmdiff, auc Z: **auc** R: start	
KFA	X: **std**, mmdiff, mmtdiff, **auc** Y: mmtdiff Z: ttmax R: start, stop	X: wmax, pmax, auc Y: std, wmax R: start, std, min	X: **std**, mmdiff Y: start, pmax, **auc** Z: start, std, auc R: wmax, auc	X: std, ttmax, auc Y: start, auc Z: start, std, wmax, pmax, pmin, **auc** R: start, mmtdiff	Range
KEM	X: **std** Y: ttmax Z: start, mmdiff R: **std**, ttmax, **pmax**, min	X: wmax Z: std, min R: start, ttmax	X: wmax, pmax, auc Y: start, wmax, auc Z: mmdiff R: ttmax, wmax, wmin	X: **std**, max Z: **std**, mmdiff R: ttmax, wmax, wmin, pmin	
KPA	X: std, ttmax Y: wmin Z: wmax, ttmin, pmin, mmdiff R: **std**, **pmax**	X: min Z: stop, std R: start	X: ttmax, auc Y: start, std, **max**, **pmax** Z: std, ttmax, pmax, wmin, pmin R: start, ttmax, ttmin, pmin	X: **std**, mmdiff Y: std Z: max, wmax	

Bolded variables are the top 5 features from each selected model based on the highest absolute value of model coefficients. For variable abbreviations, see Table A1. vGRF, peak vertical ground reaction force. KFA, peak knee flexion angle. KEM, peak internal knee extension moment. KPA, peak sagittal plane knee power absorption. RMSE, root mean squared error.

## Appendix B

While we advocate for the use of absolute measures of biomechanics as opposed to relative symmetry between limbs, we recognize that limb symmetry may be of interest. Towards these ends, we calculated the limb symmetry index (LSI) of all lab-based response variables as: (left limb/right limb) × 100. We repeated this LSI calculation for all IMU-based, model-predicted values. We then calculated the RMSE of the IMU-based LSI compared to the lab-based LSI. The mean and standard deviation of the LSI for each response variable and the RMSE values for each model can be found in Table A5. Figure A1 depicts scatter plots of the IMU-based LSIs compared to the lab-based LSI.

**Table A5:** Limb symmetry analysis.

		Model RMSE (%)			
	LSI (%)	Single Feature		Multiple Feature	
	Mean ± SD	Shank	Thigh	Accel	Accel + Gyro
vGRF	89.8 ± 19.5	15.2	17.9	14.7	14.3
KFA	102.2 ± 4.1	4.1	4.2	4.0	3.5
KEM	92.4 ± 16.8	15.8	16.3	13.9	12.7
KPA	92.3 ± 23.2	21.9	20.2	17.0	15.8

vGRF, peak vertical ground reaction force. KFA, peak knee flexion angle. KEM, peak internal knee extension moment. KPA, peak sagittal plane knee power absorption. BW, body weight. HT, height. RMSE, root mean squared error. LSI, limb symmetry index. RMSE, root mean squared error.
